# Genomic Characterization of a Halovirus Representing a Novel Siphoviral Cluster

**DOI:** 10.3390/v15061392

**Published:** 2023-06-19

**Authors:** Kaixin Diao, Guohui Li, Xueqin Sun, Hao Yi, Shiying Zhang, Wei Xiao

**Affiliations:** 1 Yunnan Institute of Microbiology, Yunnan International Joint Laboratory of Virology & Immunology, Yunnan University, Kunming 650500, China; 2 Yunnan Soil Fertilization and Pollution Remediation Engineering Research Center, Yunnan Agricultural University, Kunming 650201, China

**Keywords:** *Halomonas*, bacteriophage, genome, new genus, auxiliary metabolic genes

## Abstract

Salt mines are a special type of hypersaline environment. Current research mainly focuses on prokaryotes, and the understanding of viruses in salt mines remains limited. Understanding viruses in hypersaline environments is of great significance for revealing the formation and maintenance of microbial communities, energy flow and element cycling, and host ecological functions. A phage infecting *Halomonas titanicae* was isolated from Yipinglang Salt Mine in China, designated *Halomonas titanicae* phage vB_HtiS_YPHTV-1 (YPHTV-1). Transmission electron microscopy revealed that YPHTV-1 had an icosahedral head with a diameter of 49.12 ± 0.15 nm (n = 5) and a long noncontractile tail with a length of 141.7 ± 0.58 nm (n = 5), indicating that it was a siphovirus. The one-step growth curve showed that the burst size of YPHTV-1 was 69 plaque forming units (PFUs) cell^−1^. The genome of YPHTV-1 was 37,980 bp with a GC content of 36.2%. The phylogenetic analysis of the six conserved proteins indicated that YPHTV-1 formed a cluster with *Bacillus* phages and was separated from phages infecting *Halomonas*. The average nucleotide identity (ANI), phylogenetic, and network analyses indicated that the phage YPHTV-1 represented a new genus under Caudoviricetes. In total, 57 open reading frames (ORFs) were predicted in the YPHTV-1 genome, 30 of which could be annotated in the database. Notably, several auxiliary metabolic genes were encoded by YPHTV-1, such as ImmA/IrrE family metalloendopeptidase, mannose-binding lectin (MBL) folding metallohydrolase, M15 family of metal peptidases, MazG-like family protein, O antigen ligase, and acyltransferase. These genes potentially enabled the host bacterium to resist ionizing radiation, ultraviolet light (UV), mitomycin C, β-lactam antibiotic, high osmotic pressure, and nutritional deficiencies. These findings highlight the role of haloviruses in the life cycle of halobacteria.

## 1. Introduction

Hypersaline environments are distributed worldwide in different forms, such as salt lakes, sun-dried salt pans, and salt mines; archaea dominate hypersaline areas, but bacteria and some eukaryotes are also present [1]. The number of viruses in hypersaline environments can be 10–100 times more than that of the host [2,3], reaching 10^9^ virus-like particles (VLPs)/mL in some hypersaline water [4]. Viruses occupying hypersaline environments are known as haloviruses [5]. Compared with aquatic systems such as the ocean, there is less research on viruses in extreme environments such as hypersaline environments. Virus-like particles with various morphotypes have been described in hypersaline environments, ranging from 40 to 100 nm in diameter [6,7]. There are six main forms, comprising viruses with head-tailed (contractile) ΦCh1 [8], head-tailed (noncontractile) BJ1 [9], head-tailed (short) HSTV-1 [10], pleomorphic HRPV-1 [11], spherical SH1 [12], and spindle-shaped His1 [13]. Over 100 haloviruses have been isolated to date, most of which infect extreme halophilic archaea. Among these 100 haloviruses, only ~30 phages infect halophilic bacteria [14,15,16,17,18,19,20,21,22,23,24,25,26,27]. Currently, the research on culturable bacterial haloviruses mainly focuses on their morphological, physiological, and biochemical characteristics; research on the viral genome is still relatively scarce. Due to the small number of isolates, the genomes of only 16 bacterial haloviruses have been analyzed in detail [17,21,22,24,25,26,27], hindering our understanding of virus–host interactions. Four Marseille viruses and one *Mimivirus* isolated from a hypersaline environment in Tunisia using the halophilic amoeba as the host are the only known eukaryotic haloviruses [28].

The genus *Halomonas* is classified within the family *Halomonadaceae* and the order *Oceanospirillales* in the class *Gammaproteobacteria*. The cells are Gram-negative and non-endospore-forming rods and are halophilic or halotolerant. Some species in *Halomonas* are haloalkaliphilic or psychrotolerant [29]. The genus *Halomonas* is a common bacterial group found in hypersaline samples using culture-dependent methods. The genus *Halomonas* currently contains 121 species with validly published and correct names isolated from saline environments worldwide, including solar salt facilities, intertidal estuaries, the Dead Sea, and hypersaline lakes (https://lpsn.dsmz.de/genus/halomonas, accessed on 17 June 2023). In addition to being widely distributed in various hypersaline environments, *Halomonas* has substantial development potential due to its versatile properties, such as good emulsifying activities against the petroleum hydrocarbons and ability to produce polyhydroxybutyrate (PHB) and polyhydroxyalkanoate (PHA) [30,31].

The phage–host interactions of five myoviruses infecting *H. venusta* or *H. salina* from the Great Salt Plains in Oklahoma [32] and three temperate siphoviruses infecting *H. halophila* isolated from hypersaline soil have been characterized [33]. Moreover, a myovirus-like temperate phage has been successfully induced from ocean-isolated *H. aquamarina* using mitomycin C, and its genome has been sequenced [20]. However, to the best of our knowledge, there are no detailed characterizations to date of any virulent *Halomonas titanicae* viruses.

The hypersaline environment has a high similarity with the early Earth environment and the Martian environment [34]. Thus, viruses in hypersaline environments may comprise remnants of ancestral viruses [4], and excavating and studying the physiological and ecological functions of uncultured haloviruses in hypersaline environments may help elucidate the origin and evolution of life on Earth and provide a theoretical reference for the exploration and discovery of life on Mars.

This study isolated the first phage, YPHTV-1, infecting *H. titanicae* from a salt mine. The biological characterization and genomic analysis suggested that YPHTV-1 was distinct from currently known viruses. Interestingly, several auxiliary metabolic genes were encoded by YPHTV-1, suggesting potential phage–*Halomonas* interactions and a role for viruses in *Halomonas* metabolism and evolution. These results help elucidate the ecological functions of phages in hypersaline environments and lay the foundation for screening phage-resistant *Halomonas* strains for industrial applications.

## 2. Materials and Methods Sterile

### 2.1. Isolation of Host H. titanicae H5 and Phage YPHTV-1

Fifty-liter brine samples were collected from the brine sedimentation tank of Yipinglang Salt Mine (101°90′ E, 25°28′ N) in Yunnan Province in China in April 2010, which was brought back to the laboratory for storage at room temperature. *H. titanicae* H5 and its phage YPCBV-1 were isolated from these brine samples in 2019. Marine agar 2216 (MA, Difco) was used for the isolation of the host, while the strains were cultured in modified Luria-Bertani (MLB, NaCl 100 g/L, tryptone 8.0 g/L, yeast extract 4.0 g/L, [pH 7.2–7.6]). Strain genomic DNA extraction and PCR amplification of the 16S rRNA gene were performed as described previously [35], and sequencing was performed in Sangon, China. For phages isolation, 5 mL of brine was inoculated into 100 mL MLB broth, incubated at 30 °C, 120 rpm. After 7 days, enrichments were passed through a 0.22 µm pore size filter. One hundred microliters of the filtrate were mixed with 300 μL of the H5 bacterial suspension cultured to the logarithmic phase (OD600 = 0.5) and adsorbed at 25 °C for 20 min. Then, 4 mL of MLB semi-solid medium was added, and the mixture was poured onto a solid medium plate and incubated at 30 °C. A double-layer plate with the host bacterial medium without filtrate was prepared as a control [36]. After 24 h, the plaques appeared, and a large and clear single plaque was picked and transferred to 100 μL of MLB. The double-layer plate experiment was repeated five times to purify the phage. The purified phage was stored in 15% glycerol at −80 °C. The phage lysate can also be stored at 4 °C. For prepared fresh phage suspension, an appropriate amount of phages was mixed with the host, shaking at 30 °C and 120 rpm for 24 h and filtering at 0.22 µm pore size filter.

### 2.2. Transmission Electron Microscopy

The phage lysate was stained with 2% (*w*/*v*) sodium phosphotungstate (Sangon, China) for 3 min, air dried, and placed under transmission electron microscopy (TEM, JEM-2100, 200 kV) to determine the morphologies of the phage virions. Digital images were captured with a bottom mounted Quemesa camera and analyzed using the iTEM software (v5.2).

### 2.3. Host Range

The host range of YPHTV-1 was assessed using nine different bacteria: *Chromohalobacter japonicus* (CGMCC 1.7474), *C. canadensis* (CGMCC 1.7979), *C. marismortui* (CGMCC 1.2321), and *C. beijerinckii* (CGMCC 1.9020) purchased from China General Microbiological Culture Collection Center (CGMCC). *C. beijerinckii* F3, *C. canadensis* F7, *H. titanicae* H5, *H. ventosae* QH52-2, and *H. titanicae* H5G were isolated from salt mine samples in our laboratory. The phage specificity was determined by dropping 10 μL of YPHTV-1 lysate onto lawns of the aforementioned nine strains. Plaque formation was observed after incubating at 30 °C for 24 h.

### 2.4. One-Step Growth Curve

A one-step growth assay was performed as previously described [36]. Briefly, 1 mL of the host culture (7.2 × 10^7^ CFU/mL) was mixed with the phage lysate at the optimal MOI (=1) and adsorbed at 30 °C for 20 min. The sample was centrifuged at 10,000× *g* for 5 min, the supernatant was discarded, and the pellet was resuspended in 1 mL MLB medium. This step was repeated twice to remove unabsorbed phage particles. The precipitate was transferred to 50 mL of MLB medium and incubated at 30 °C, 120 rpm for 2 h. The phage titer in the culture was measured using the double-layer agar technique at 10 min intervals. The burst size was calculated as follows: ($\frac{\mathrm{phage}\;\mathrm{titer}\;\mathrm{at}\;\mathrm{the}\;\mathrm{end}\;\mathrm{of}\;\mathrm{the}\;\mathrm{burst}}{\mathrm{initial}\;\mathrm{host}\;\mathrm{cell}\;\mathrm{concentration}}$) ×100%. All experiments were performed in triplicate.

### 2.5. pH and Thermal Stability

The pH and thermal stability analyses were performed as previously described [36]. To study the effect of pH on phage survival, Tris-HCl buffer (0.1 M) and NaOH solution (0.1 M) were used to adjust the pH of the liquid MLB medium to pH 3, 4, 5, 6, 7, 8, 9, 10, 11, and 12. Phage lysate was added to different pH solutions and inoculated at room temperature for 1 h. One milliliter of phage lysate was inoculated and incubated for 1 h at 22 °C, 30 °C, 37 °C, 50 °C, and 60 °C to study the thermal stability of the phage. The phage titers were measured by the double-layer agar plate method at the indicated times. The phage titer measured at 0 min was used as a control to calculate the survival rate. All experiments were performed in triplicate.

### 2.6. Phage Adsorption Rate to Different Cells

*H. titanicae* H5, *H. titanicae* H5G, *H. ventosae* QH52-2, *C. beijerinckii* F3, and *C. canadensis* F7 were cultured to the logarithmic phase (OD_600_ = 0.5). One hundred microliters of the phage lysate were mixed with 300 μL of different bacterial cultures, adsorbed at room temperature for 20 min, and then centrifuged at 10,000× *g* for 5 min. The phage titers in the supernatant were tested using the titer of the initial phage lysate as a control. The adsorption rate of the phage to different cells was calculated as follows: ($\frac{\mathrm{titer}\;\mathrm{of}\;\mathrm{initial}\;\mathrm{phage}\;\mathrm{lysate}-\mathrm{titer}\;\mathrm{of}\;\mathrm{unadsorbed}\;\mathrm{phage}\;\mathrm{lysate}\;}{\mathrm{titer}\;\mathrm{of}\;\mathrm{initial}\;\mathrm{phage}\;\mathrm{lysate}}$) × 100%. All experiments were performed in triplicate. ANOVA analysis was used to test the difference in the adsorption rate.

### 2.7. Preparation of High-Titer Phage Lysate

A sterile bamboo stick was used to pick and purify a single plaque and place it in 100 μL of SW buffer with an NaCl concentration of 5% (*w*/*v*), and 1 mL of the host bacteria H5 suspension cultured to the logarithmic phase was added and incubated at 120 rpm, 30 °C for 12 h. After the culture solution was centrifuged at 11,000× *g* at 4 °C for 25 min, the supernatant was passed through a 0.22 µm pore size filter to obtain a phage suspension. The double-layer agar plate method was used to spread the phage suspension onto 10 plates. When the plaque formed, the upper agar with the plaque was scraped and inoculated into 100 mL of the host bacterial suspension and incubated at 120 rpm, 30 °C, for 12 h. The phage-enriched liquid was collected and centrifuged at 11,000× *g* for 20 min at 4 °C, and the supernatant was passed through a 0.22 µm pore size filter to obtain a high-titer phage lysate.

### 2.8. Extraction, Sequencing, and Bioinformatic Analysis of Phage DNA

The high-titer phage lysate was added to a 100 KD ultrafiltration tube (Millipore, Burlington, MA, USA) and centrifuged at 4500× *g* and 4 °C to obtain the phage concentrate. DNase I and RNase A (Solarbio, Beijing, China) were added to 200 μL phage concentrate to a final concentration of 50 U/mL and 250 μg/mL, respectively, treated at 37 °C for 1 h, and inactivated at 80 °C for 15 min. The phage DNA was extracted using a TIANamp Virus DNA/RNA Kit (TIANGEN, Beijing, China) according to the manufacturer’s instructions. The DNA library was constructed as previously described [27] and was sent to Sangon Bioengineering Co., Ltd., for sequencing using Illumina HiSeq. After sequencing, the original data were evaluated and quality controlled. Clean data were assembled into a single contig using SPAdes (v3.5.0) [37], and the gaps were filled using GapFiller (v 1.11) [38]. The ORFs were predicted using GeneMarks (v.3.26) [39]. The BLAST program on the NCBI website (http://www.ncbi.nlm.nih.gov/, accessed on 19 May 2023) was used to find similar sequences to each of the ORFs in YPHTV-1. The genome network of YPHTV-1 was analyzed using the Prokaryotic Viral RefSeq211 Merged (last updated in June 2022) database of vConTACT (v.2.0) [40], and Cytoscape [41] software was used to create a network map for visualization. The VIRIDIC [42] tool was used to calculate the intergenomic similarities of viruses. The amino acid sequences of HNH endonuclease, terminal enzyme large subunit (TerL), portal protein, major capsid protein, and MazG were selected to construct a phylogenetic tree using the Mega 7.0 software package with the neighbor-joining method. The phylogenetic tree of the phage genome was constructed using ViPTree (v3.5) (https://www.genome.jp/viptree/, accessed on 19 May 2023) [43]. Ezbiocloud (https://www.ezbiocloud.net/tools/ani, accessed on 10 December 2022) was used to perform the ANI pairwise comparison of 24 phage genome sequences, and ANI heat maps were generated using R studio (v 1.3.1093). EasyFig (v2.2.3) [44] was used to compare highly homologous genomes.

## 3. Results

### 3.1. Biological Characteristics of YPHTV-1

**Figure 1:**
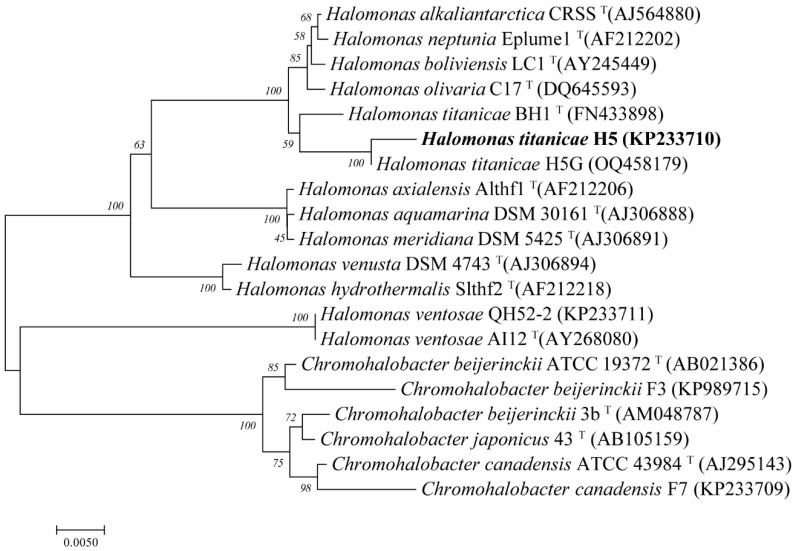
Phylogenetic analysis of H5 based on the 16S rRNA gene sequence. Phylogenetic trees were constructed using the neighbor-joining method by Mega 7.0. All parameters were default except the Bootstrap value was 1000, the p-distance model was used to calculate the distance, and the Gap/Missing Data Treatment cutoff was 50%.

The 16S rRNA gene phylogenetic analysis of strain H5 isolated from Yipinglang Salt Mine showed that it was clustered with *H. titanicae* BH1 (KY471040), with a similarity of 98.33%. Moreover, H5 and H5G were clustered together with a similarity of 99.93% (Figure 1). Thus, it was preliminarily identified that the strain H5 belonged to *Halomonas titanicae*.

**Figure 2:**
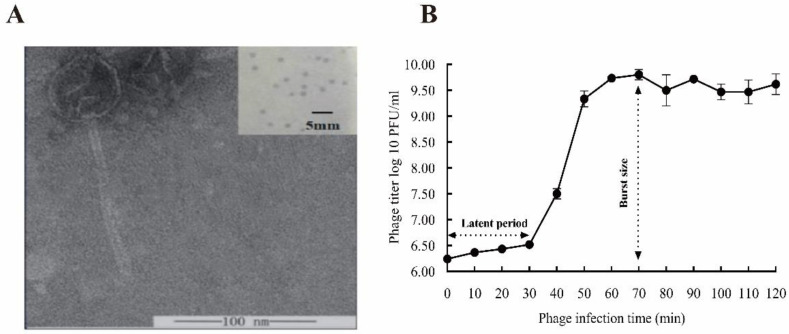
Biological features of phage YPHTV-1. (**A**) Transmission electron micrograph of YPHTV-1. Scale bar, 100 nm. Inset shows plaques morphology of YPHTV-1; scale bar, 5 mm. (**B**) One-step growth curve of phage YPHTV-1. Error bars represent the standard deviation of three replicates.

The lytic phage YPHTV-1 was isolated from the brine collected from Yipinglang Salt Mine using *H. titanicae* H5 as the host. After 24 h, clear circular plaques formed, 1.5–2.0 mm in diameter, surrounded by a cloudy halo (Figure 2A). TEM showed that phage YPHTV-1 had an icosahedral head with a diameter of 49.12 ± 0.15 nm (n = 5) and a long tail 141.7 ± 0.58 nm (n = 5) in length (Figure 2A), indicating that it was a siphovirus. The full infection cycle of YPHTV-1 was 70 min, of which the latent period was 30 min and the rapid growing period was 40 min; the burst size was 69 PFU/cell (Figure 2B).

**Figure 3:**
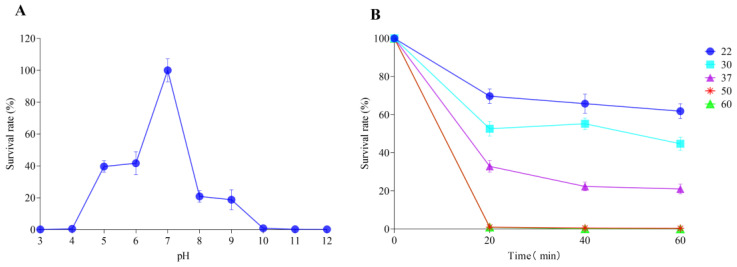
The pH (**A**) and thermal (**B**) stability of YPHTV-1. Error bars represent the standard deviation of three replicates.

YPHTV-1 was most stable at pH 7.0. When the pH was lower than 5 or higher than 9, the survival rate of the phage YPHTV-1 dropped to close to 0 (Figure 3A). After incubation at 22 °C, 30 °C, and 37 °C for 20 min, the survival rate of the phage YPHTV-1 slightly decreased. After incubation at 50 °C and 60 °C for 20 min, the survival rate of the phage YPHTV-1 dropped to 0 (Figure 3B). These findings show that the phage YPHTV-1 is stable in a neutral environment and at 22–37 °C.

A cross-infectivity test was performed to examine the host range of YPHTV-1 within an extensive collection of nine strains of species of the family *Halomonadaceae*. The phage YPHTV-1 only infected its host strain, H5; even H5G, isolated from Qiaohou Salt Mine and sharing high 16S rRNA gene sequence similarity with H5, was not infected by YPHTV-1 (Figure 1).

**Figure 4:**
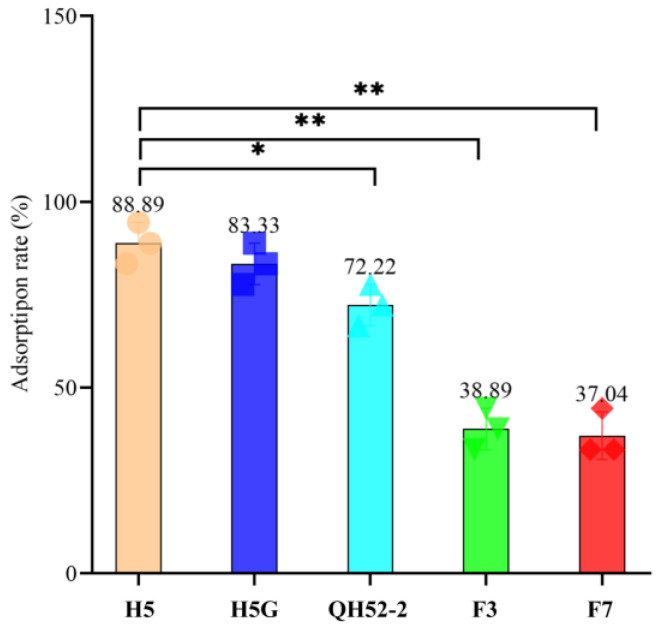
The adsorption of phage YPHTV-1 to different cells. H5 and H5G belong to *H. titanicae*, QH52-2 belongs to *H. ventosae*, F3 belongs to *C. beijerinckii*, and F7 belongs to *C. canadensis.* The numerical value represents the adsorption rate of the virus on different cells. ANOVA analysis was used to test the difference in the viral adsorption rate between the host bacterium H5 and other cells (* 0.01 < *p* < 0.05, ** *p* < 0.01).

The first step of infection was identification and adsorption. Phage YPHTV-1 had the highest adsorption rate to strain H5 and H5G. The adsorption rate to *H. ventosae* QH52-2 was significantly reduced, and this reduction was more pronounced in *C. beijerinckii* F3 and *C. canadensis* F7 (Figure 4). These results suggested that the inability of the phage YPHTV-1 to infect the H5G was caused by the steps after adsorption.

### 3.2. Genomic Property of Phage YPHTV-1

**Figure 5:**
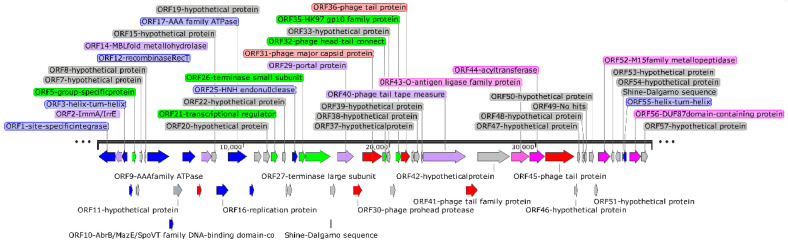
Genome map of YPHTV-1. The arrows represent ORFs, and different colors represent different functions. Red, structural protein; green, packaging transcription protein; blue, DNA replication, regulation, and nucleotide metabolism protein; purple, lytic and lysogenic protein; pink, other functional proteins; and gray, hypothetical protein with unknown function.

Ninety-nine bp direct repeats were identified at both ends of the genome, indicating that YPHTV-1 was linear double-stranded DNA. The sequence was found to be 37,980 bp in length with a GC content of 36.2%. GeneMark predicted 57 ORFs in the YPHTV-1 genome, 51 in the positive strand, and six in the negative strand (Figure 5). Among them, 30 were assigned a putative function based on significant sequence similarity to genes of known functionality in the NR database. Furthermore, 27 ORFs showed no similarity to genes in the NR database, and their products were classified as hypothetical proteins with unknown functions (Table S1). Potential tRNAs were not detected in the phage genome.

The YPHTV-1 genome exhibited an overall modular organization (Figure 5). The functional gene modules annotated in the YPHTV-1 genome and their proportions of the total number of genes were as follows: lytic/lysogenic proteins (14%), structural protein (14%), DNA replication/regulation and nucleotide metabolism proteins (19%), packaging proteins and transcription-related proteins (12%), and other functional genes (10%).

### 3.3. DNA Replication and Virion Assembly

ORF40 encoded the phage tail tape measure protein (TMP), a protein that played a key role in phage DNA injection and was related to the host super-infection immunity. ORF16 encoded the phage replication protein. The replication protein has been shown to increase the rate of viral DNA replication [45]. The protein encoded by ORF25 belonged to the HNH endonuclease superfamily. Most HNH endonucleases contained a conserved HNH catalytic center and a zinc ion binding site [CXXC]_2_, which, in the presence of divalent metal ions, could cut 3–5 bp double-stranded DNA [46]. Therefore, HNH endonuclease was a critical assembly machine in the phage life cycle and was of great significance to phage reproduction and infection [47,48]. The packaging module of phage YPHTV-1 included a terminase and a portal protein. ORF26 and ORF27 encoded the terminase small and large subunits, respectively, and were involved in packaging the phage genome into the capsid [48]. ORF30 encoded the phage YPHTV-1 prohead protease. This gene was usually located near the capsid protein gene (ORF31). In some cases, these two genes were fused [49].

### 3.4. Auxiliary Metabolic Genes

ORF2 encoded the ImmA/IrrE family metalloendopeptidase. ImmA-dependent proteolysis of ImmR repressors may be a conserved mechanism for regulating horizontal gene transfer [50] and radiation resistance [51,52]. ORF14 putatively encoded the MBL folding metallohydrolase belonging to the metalloenzyme superfamily I, potentially improving antibiotic resistance [53]. ORF18 encoded the MazG-like family protein. MazG potentially optimized the progeny phage production by reactivating macromolecule synthesis pathways [54]. ORF43 encoded the O antigen ligase. O antigen ligase participated in the synthesis of lipopolysaccharide (LSP), protected the host from other bacteria or viruses, changed the permeability of cell membrane, tolerated high osmotic pressure, adapted to high-salt environments, increased the resistance of the host, and improved the adaptability of the host to extreme environments [55].

### 3.5. Phage YPHTV-1 Represents a New Cluster

**Figure 6:**
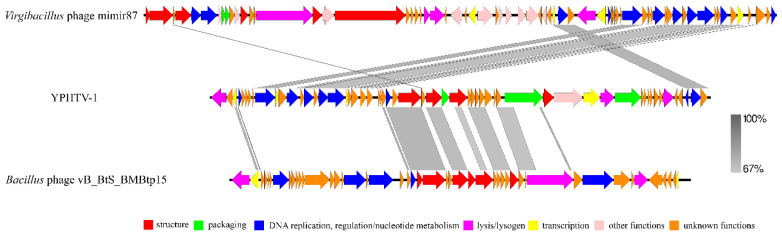
Genome comparison between YPHTV-1 and *Virgibacillus* phage Mimir87 and *Bacillus* phage vB_BtS_BMBtp15. The arrow indicates the direction of gene transcription. The gray bar represents similarity.

BLAST showed that the genome sequence of YPHTV-1 was not significantly similar to other viruses, and only a few viruses had partial sequence similarity. The similarity between YPHTV-1 and the *Virgibacillus* phage Mimir 87 (MK560763) was 70.08% (coverage is 22%). The phage Mimir 87 was isolated from worker bees and infected salt-tolerant *Bacillus* sp. The similar regions of YPHTV-1 and Mimir 87 were genes encoding DNA replication/regulation and nucleotide metabolism and transcription (DNA replication, recombinase protein RecT, and AAA family ATPase) (Figure 6). In addition, the similarity between YPHTV-1 and the *Bacillus* phage vB_BtS_BMBtp15 (KX190835.1) was 72.80% (coverage was 19%). The similar regions of YPHTV-1 and vB_BtS_BMBtp15 were genes encoding packaging and morphological structure (portal protein, terminase, and capsid family protein) (Figure 6).

**Figure 7:**
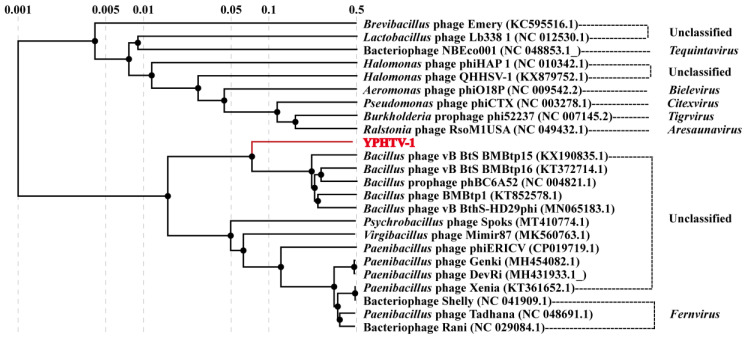
Phylogenetic analysis of YPHTV-1 based on the genome sequences. Phylogenetic trees were constructed using the ViPTree. All parameters were default except the analysis where the reference was “NO”.

Twenty-three viral genome sequences in GenBank (six with established taxonomic status in ICTV) were compared with the YPHTV-1 to further clarify its evolutionary status. A total of 24 phages were used to construct a phylogenetic tree with amino acid sequences and a heat map with genome-wide ANI. The phylogenetic analysis showed that the phage YPHTV-1 was clustered with an unclassified *Bacillus* phage (Figure 7). The ANI values of YPHTV-1 in comparison with 23 other viral genomes were 0–70.85% (Figure S1), among which the ANI values of the *Bacillus* phages were higher (*Bacillus* phage vB_BtS_BMBtp15 was 70.23%; *Bacillus* phage vB_BtS_BMBtp1 was 70.85%).

**Figure 8:**
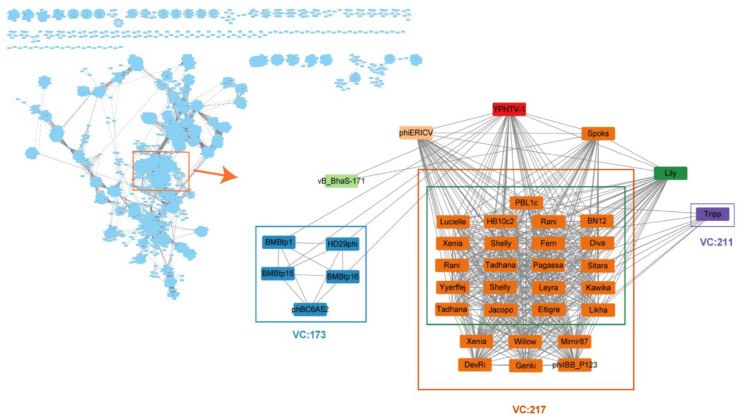
YPHTV-1 genome network analysis. Genomic network analysis was performed by vConTACT2, ClusterONE, and Cytoscape. The reference database was Prokaryotic Viral RefSeq211-Merged (last updated in June 2022), and virus clusters are shown as differently colored boxes. VC217 (orange box): most of the phages were infected with *Paenibacillus* and belonged to the *Fernvirus* genus of Caudoviricetes (green box in VC 217); VC173 (blue box): infected with *Bacillus* with no classification status determined; VC211 (purple box): belonged to *Halcyonevirus* genus of Caudoviricetes. The classification status of viruses was derived from the ICTV.

YPHTV-1 ORF25 (HNH endonuclease), ORF27 (terminase large subunit), ORF29 (portal protein), ORF31 (major capsid protein), ORF1 (integrase), and ORF18 (MazG-like family protein) were selected for the phylogenetic analysis. The phylogenetic tree of the five proteins showed that YPHTV-1 clustered with the *Bacillus* phages (Figure S2), consistent with the genome-wide analysis. It was demonstrated that YPHTV-1 shared homology with the *Bacillus* phages in integrase, packaging, and structural proteins and shared homology with the *Paenibacillus* phages in the MazG-like family protein. *Bacillus* was also a predominant microbe in hypersaline environments and has been found in 220-million-year-old salt crystals [56]. The genome network analysis showed that 37 viruses were related with phage YPHTV-1, but phage YPHTV-1 did not cluster with any viruses (Figure 8). Thirty-three out of the 37 viruses were divided into three clusters, 27 phages in VC217, five phages in VC173, and one phage in VC211. The four independent phages, VC173 and VC211, had no definite classification status in ICTV, while almost all viruses in VC217 infected *Paenibacillus* and belonged to the *Fernvirus* genus under *Caudoviricetes* (Figure 8, shown in the green box). The VIRIDIC analysis showed that among the three virus clusters, VC173 had the highest average similarity with YPHTV-1 (13.66%); similarity was also found with the *Bacillus* phage vB_BtS_BMBtp15 (14.54%) and vB_BthS-HD29phi (14.13%) in VC173, while that with VC217 was only 3.90%. This finding was consistent with the results of ANI analysis, indicating that YPHTV-1 clustered with viruses infecting *Bacillus* and *Paenibacillus*. Notably, except for the *Bacillus cereus* phage phBC6A52, YPHTV-1 and the other four viruses in VC173 were siphoviruses [57,58]. However, the affinity was insufficient to support that the phage YPHTV-1 belonged to this currently known virus genus; YPHTV-1 may represent a new virus genus.

The phylogenetic, ANI comparison, and genome network analyses all suggested that the phage YPHTV-1 was related to *Bacillus* phages. The morphology of the phage showed that it was a siphovirus. We suggest the phage YPHTV-1 represents a new genus under the class Caudoviricetes.

## 4. Discussion

Viruses are thought to be the most abundant biological entities on Earth; however, very few have been cultivated. There remain numerous viruses that have yet to be recognized. In this study, we isolated a new siphovirus, YPHTV-1, infecting *H. titanicae* H5, from salt mine samples stored at room temperature for 10 years. YPHTV-1 was very stable at room temperature and in a hypersaline environment, and its genome contained genes that may promote the host’s resistance to stress.

YPHTV-1 had a high specificity for the host, which has also been observed in the halovirus CGΦ29 [22]. The first step of phage infection is recognition and adsorption. Bacteria can prevent phage attachment by lacking or hiding surface receptors. Even if phages attach to appropriate surface receptors successfully, a superinfection rejection system and other mechanisms can prevent phage DNA from being injected into host cells [59]. After a phage has injected its DNA into bacterial cells, the natural defense system in the host cells can also prevent phage proliferation, such as abortive infection and restriction modification systems that can cause the host to cut or degrade phage DNA before releasing the progeny virus, preventing phage replication and release [60]. These influencing factors also determine the host range of phages. The inability of the phage YPHTV-1 to infect the H5G was caused by the steps after adsorption.

To date, genome sequencing has been completed for two viruses infecting *Halomonas* sp., i.e., the myoviral phage ΦHAP-1 induced by mitomycin C from the surface water of the Gulf of Mexico [20] and the siphoviral phage QHHSV-1 isolated from Qiaohou Salt Mine in China [25]. These three *Halomonas* phages have similar genome sizes (37,270–39,245 bp). The phage YPHTV-1 has the lowest GC content among the three *Halomonas* phages (36.2% (YPHTV-1) vs. 59% (ΦHAP-1) and 66.8% (QHHSV-1)).

One mechanism by which phages alter the metabolic state of the host is through the activity of phage-encoded AMGs [61,62]. AMGs are typically obtained from host cells (i.e., recombined onto phage genomes) and can enhance or redirect specific metabolic processes within host cells during infection [63,64]. These enhancements may play an important role in maintaining, driving, or shortening the metabolic pathways and may provide phages and their hosts with sufficient fitness advantages to retaining these genes over time under specific metabolic or nutritional conditions [65]. Many AMGs were predicted in the YPHTV-1 genome, and their functions involved radiation resistance (*ImmA*/*IrrE* family metalloendopeptidase), antibiotic resistance (MBL folding metallohydrolase), regulation of osmotic pressure (O antigen ligase), and response to starvation (MazG-like family protein).

Ludanyi et al. have found that ImmA/IrrE was required for *Deinococcus* resistance to ionizing radiation, ultraviolet light, and mitomycin C as a global transcriptional regulator [51]. The MBL folding metallohydrolase can be combined with cysteine, histidine, aspartic acid, and other active sites of amino acids and zinc and then combined with water molecules for activation, after which it becomes a nucleophile, with a wide range of hydrolysis effects on β-lactam antibiotics, leading to resistance to cephalosporins, carbapenems, and other antibiotics and playing an important role in the bacterial carbon-phosphorus lyase (CP-Lyase) pathway [53]. The O antigen ligase connection of O antigen to the core region of the lipid A core is an important step in the LPS biosynthetic pathway. O antigen ligase and acyltransferase participate in the synthesis of LSP to protect the host from other bacteria or viruses, which can change the cell membrane permeability, increase tolerance to high osmotic pressure, cause adaptation to hypersaline environments, and increase antibiotic resistance, improving the ability of the host to adapt to extreme environments [66]. MazG is considered a regulator of programmed cell death in *E. coli*. MazG prevents the normal accumulation of guanosine 3’, 5’-dipyrophosphate (ppGpp) during the stringent response to amino acid starvation, thereby enhancing host survival under nutrient-depleted environments [67]. Therefore, it has long been held that *mazG*-carrying phages may modulate the metabolism of host cells during infection to ensure a sufficient proliferation of progeny virions [68]. The T5 phage encodes a specific product containing the M15 metallopeptidase domain, which is related to phage endolysin [69]. Researchers have found that the D-alanyl-D-alanine carboxypeptidase domain in the LysECD7 gene of *Acinetobacter* phage AM24 belongs to the M15 family of peptidases. LysECD7 contains two of these residues (Trp80 and Trp105) containing an 8-His tag sequence at the C terminus, and adjacent sequences form a positively charged cluster that increases membrane permeability [70]. In conclusion, M15 family peptidases are a very large gene family that is particularly important for both the phage and the host. On one hand, the M15 family peptidases are related to endolysin synthesis and can improve the lysis efficiency of lytic phages. On the other hand, certain domains of M15 family peptidases maintain membrane permeability, which is beneficial for host survival in hypersaline environments. These predictions need to be backed up by more experimental results.

BLAST showed that the genome of YPHTV-1 was not significantly similar to that of other known viruses. The ANI and phylogenetic analyses showed that YPHTV-1 had lower genomic similarities and genetic relationships with phages infecting *Virgibacillus*, *Bacillus,* and *Paenibacillus*. The genomic network analysis revealed that YPHTV-1 had no evolutionary relationship with the two other sequenced *Halomanas* phages but clustered with *Bacillus* and *Paenibacillus* phages with low similarity (13.54%). Therefore, YPHTV-1 is a new genus under the class Caudoviricetes.

At present, there is insufficient research on haloviruses, and little is known about their genomes. This study analyzed the biological characteristics and genome of the halovirus YPHTV-1, providing a basis for the follow-up study of the phage–host interaction.

## Data Availability

The genome sequence of phage YPHTV-1 and the 16S rRNA gene sequence of *Halomonas titanicae* H5 have been deposited in the GenBank under accession numbers ON854455 and KP233710, respectively.

## Supplementary Materials

The following supporting information can be downloaded at https://www.mdpi.com/article/10.3390/v15061392/s1: Figure S1: Phage genome ANI heat map. The horizontal and vertical coordinates are the phage names, and the color of the intersecting squares represents the ANI value of the two phages; Figure S2: The amino acid sequence phylogenetic trees based on HNH endonuclease (A), terminase large subunit (B), portal protein (C), major capsid protein (D), site-specific integrase (E), and MazG-like family protein (F) of YPHTV-1 and other phages. Phylogenetic trees were constructed using the neighbor-joining method by Mega 7.0. All parameters were default except the bootstrap value was 1000, the p-distance model was used to calculate the distance, and the Gap/Missing Data Treatment cutoff was 50%; Table S1: Gene annotation of phage YPHTV-1 (E < 10^−5^).

## References

[1] Oren A. (2008). Microbial life at high salt concentrations: Phylogenetic and metabolic diversity. Saline Syst..

[2] Rodriguez-Valera F., Martin-Cuadrado A.-B., Rodriguez-Brito B., Pasic L., Thingstad T.F., Rohwer F., Mira A. (2009). Explaining microbial population genomics through phage predation. Nat. Rev. Microbiol..

[3] Narasingarao P., Podell S., Ugalde J.A., Brochier-Armanet C., Emerson J.B., Brocks J.J., Heidelberg K.B., Banfield J.F., Allen E.E. (2012). De novo metagenomic assembly reveals abundant novel major lineage of Archaea in hypersaline microbial communities. ISME J..

[4] Atanasova N.S., Oksanen H.M., Bamford D.H. (2015). Haloviruses of archaea, bacteria, and eukaryotes. Curr. Opin Microbiol..

[5] Atanasova N.S., Bamford D.H., Oksanen H.M. (2016). Virus-host interplay in high salt environments. Environ. Microbiol. Rep..

[6] Boujelben I., Yarza P., Almansa C., Villamor J., Maalej S., Antón J., Santos F. (2012). Virioplankton Community Structure in Tunisian Solar Salterns. Appl. Environ. Microbiol..

[7] Garcia-Heredia I., Martin-Cuadrado A.-B., Mojica F.J.M., Santos F., Mira A., Antón J., Rodriguez-Valera F. (2012). Reconstructing Viral Genomes from the Environment Using Fosmid Clones: The Case of Haloviruses. PLoS ONE.

[8] Witte A., Baranyi U., Klein R., Sulzner M., Luo C., Wanner G., Kru¨ger D.H., Lubitz W. (1997). Characterization of Natronobacterium magadii phage φCh1, a unique archaeal phage containing DNA and RNA. Mol. Microbiol..

[9] Pagaling E., Haigh R.D., Grant W.D., Cowan D.A., Jones B.E., Ma Y., Ventosa A., Heaphy S. (2007). Sequence analysis of an Archaeal virus isolated from a hypersaline lake in Inner Mongolia, China. BMC Genom..

[10] Pietilä M.K., Laurinmäki P., Russell D.A., Ko C.-C., Jacobs-Sera D., Hendrix R.W., Bamford D.H., Butcher S.J. (2013). Structure of the archaeal head-tailed virus HSTV-1 completes the HK97 fold story. PNAS.

[11] Pietilä M.K., Roine E., Paulin L., Kalkkinen N., Bamford D.H. (2009). An ssDNA virus infecting archaea: A new lineage of viruses with a membrane envelope. Mol. Microbiol..

[12] Dyall-Smith M., Tang S.-L., Bath C. (2003). Haloarchaeal viruses: How diverse are they?. Res. Microbiol..

[13] Pietilä M.K., Atanasova N.S., Oksanen H.M., Bamford D.H. (2013). Modified coat protein forms the flexible spindle-shaped virion of haloarchaeal virus His1. Environ. Microbiol..

[14] Calvo C., García de la Paz A., Pérez-Martínez A., Ramos-Cormenzana A. (1991). Isolation of phages HM5 and HM15 from hypersaline soil. Toxicol. Environ. Chem..

[15] Kauri T., Ackermann H.-W., Goel U., Kushner D.J. (1991). A bacteriophage of a moderately halophilic bacterium. Arch. Microbiol..

[16] Calvo C., Paz A.M.G.D.L., Caba F.M.C.M.A. (1994). Behaviour of two *D. halophila* bacteriophages with respect to salt concentrations and other environmental factors. Toxicol. Environ. Chem..

[17] Villamor J., Ramos-Barbero M.D., González-Torres P., Gabaldón T., Rosselló-Móra R., Meseguer I., Martínez-García M., Santos F., Antón J. (2018). Characterization of ecologically diverse viruses infecting co-occurring strains of cosmopolitan hyperhalophilic Bacteroidetes. ISME J..

[18] Wang C.-X., Li X. (2018). JMT-1: A novel, spherical lytic halotolerant phage isolated from Yuncheng saline lake. Braz. J. Microbiol..

[19] Wang C.-X., Zhao A.-H., Yu H.-Y., Wang L.-L., Li X. (2022). Isolation and Characterization of a Novel Lytic Halotolerant Phage from Yuncheng Saline Lake. Indian J. Microbiol..

[20] Mobberley J.M., Authement R.N., Segall A.M., Paul J.H. (2008). The Temperate Marine Phage ΦHAP-1 of *Halomonas aquamarina* Possesses a Linear Plasmid-Like Prophage Genome. J. Virol..

[21] Olonade I., van Zyl L.J., Trindade M. (2021). Genomic characterization of a prophage, Smhb1, that infects *Salinivibrio kushneri* BNH isolated from a namib desert saline spring. Microorganisms.

[22] Rodela M.L., Sabet S., Peterson A., Dillon J.G. (2019). Broad environmental tolerance for a Salicola host-phage pair isolated from the Cargill Solar Saltworks, Newark, CA, USA. Microorganisms.

[23] Aalto A.P., Bitto D., Ravantti J.J., Bamford D.H., Huiskonen J.T., Oksanen H.M. (2012). Snapshot of virus evolution in hypersaline environments from the characterization of a membrane-containing *Salisaeta* icosahedral phage 1. Proc. Natl. Acad. Sci. USA.

[24] Shen P.S., Domek M.J., Sanz-García E., Makaju A., Taylor R.M., Hoggan R., Culumber M.D., Oberg C.J., Breakwell D.P., Prince J.T. (2012). Sequence and Structural Characterization of Great Salt Lake Bacteriophage CW02, a Member of the T7-Like Supergroup. J. Virol..

[25] Fu C., Zhao Q., Li Z., Wang Y., Zhang S., Lai Y., Xiao W., Cui X. (2017). Complete genome sequence of *Halomonas ventosae* virulent halovirus QHHSV-1. Arch. Virol..

[26] Zrelovs N., Cernooka E., Dislers A., Kazaks A. (2020). Isolation and characterization of the novel Virgibacillus-infecting bacteriophage Mimir87. Arch. Virol..

[27] Yi H., Fu C., Diao K., Li Z., Cui X., Xiao W. (2022). Characterization and genomic analysis of a novel halovirus infecting Chromohalobacter beijerinckii. Front. Microbiol..

[28] Boughalmi M., Saadi H., Pagnier I., Colson P., Fournous G., Raoult D., La Scola B. (2013). High-throughput isolation of giant viruses of the Mimiviridae and Marseilleviridae families in the Tunisian environment. Environ. Microbiol..

[29] Whitman W.B., Rainey F., Kämpfer P., Trujillo M., Chun J., DeVos P. (2015). Halomonas. Bergey’s Manual of Systematics of Archaea and Bacteria.

[30] Chen Z., Wan C. (2017). Non-sterile fermentations for the economical biochemical conversion of renewable feedstocks. Biotechnol. Lett..

[31] Tan D., Xue Y.-S., Aibaidula G., Chen G.-Q. (2011). Unsterile and continuous production of polyhydroxybutyrate by Halomonas TD01. Bioresour. Technol..

[32] Seaman P.F., Day M.J. (2007). Isolation and characterization of a bacteriophage with an unusually large genome from the Great Salt Plains National Wildlife Refuge, Oklahoma, USA. FEMS Microbiol. Ecol..

[33] Calvo C., De La Paz A.G., Béjar V., Quesada E., Ramos-Cormenzana A. (1988). Isolation and characterization of phage F9-11 from a lysogenicDeleya halophila strain. Curr. Microbiol..

[34] Parro V., de Diego-Castilla G., Moreno-Paz M., Blanco Y., Cruz-Gil P., Rodríguez-Manfredi J.A., Fernández-Remolar D., Gómez F., Gómez M.J., Rivas L.A. (2011). A Microbial Oasis in the Hypersaline Atacama Subsurface Discovered by a Life Detector Chip: Implications for the Search for Life on Mars. Astrobiology.

[35] Xiao W., Wang Y.-X., Liu J.-H., Wang Z.-G., Zhang X.-X., Ji K.-Y., Lai Y.-H., Wen M.-L., Cui X.-L. (2012). *Roseivivax sediminis* sp. nov., a moderately halophilic bacterium isolated from salt mine sediment. Int. J. Syst. Evol. Microbiol..

[36] Fu C.-Q., Zhao Q., Li Z.-Y., Wang Y.-X., Zhang S.-Y., Lai Y.-H., Xiao W., Cui X.-L. (2015). A novel Halomonas ventosae-specific virulent halovirus isolated from the Qiaohou salt mine in Yunnan, Southwest China. Extremophiles.

[37] Bankevich A., Nurk S., Antipov D., Gurevich A.A., Dvorkin M., Kulikov A.S., Lesin V.M., Nikolenko S.I., Pham S., Prjibelski A.D. (2012). SPAdes: A new genome assembly algorithm and its applications to single-cell sequencing. J. Comput. Biol..

[38] Boetzer M., Pirovano W. (2012). Toward almost closed genomes with GapFiller. Genome Biol..

[39] Besemer J., Alexandre L., Borodovsky M. (2001). GeneMarkS: A self-training method for prediction of gene starts in microbial genomes. Implications for finding sequence motifs in regulatory regions. Nucleic Acids Res..

[40] Bin Jang H., Bolduc B., Zablocki O., Kuhn J.H., Roux S., Adriaenssens E.M., Brister J.R., Kropinski A.M., Krupovic M., Lavigne R. (2019). Taxonomic assignment of uncultivated prokaryotic virus genomes is enabled by gene-sharing networks. Nat. Biotechnol..

[41] Shannon P., Markiel A., Ozier O., Baliga N.S., Wang J.T., Ramage D., Amin N., Schwikowski B., Ideker T. (2003). Cytoscape: A software environment for integrated models of Biomolecular Interaction Networks. Genome Res..

[42] Moraru C., Varsani A., Kropinski A.M. (2020). VIRIDIC—A Novel Tool to Calculate the Intergenomic Similarities of Prokaryote-Infecting Viruses. Viruses.

[43] Nishimura Y., Yoshida T., Kuronishi M., Uehara H., Ogata H., Goto S. (2017). ViPTree: The viral proteomic tree server. Bioinformatics.

[44] Sullivan M.J., Petty N.K., Beatson S.A. (2011). Easyfig: A genome comparison visualizer. Bioinformatics.

[45] Carrascosa J.L., Camacho A., Moreno F., Jimenez F., Mellado R.P., Viñuela E., Salas M. (1976). Bacillus subtilis Phage phi 29 Characterization of Gene Products and Functions. JBIC J. Biol. Inorg. Chem..

[46] Cheng H., Li M., Zhao C., Yang K., Li K., Peng M., Yang Z., Liu F., Liu Y., Bai R. (2015). Concentrations of toxic metals and ecological risk assessment for sediments of major freshwater lakes in China. J. Geochem. Explor..

[47] Kala S., Cumby N., Sadowski P.D., Hyder B.Z., Kanelis V., Davidson A.R., Maxwell K.L. (2014). HNH proteins are a widespread component of phage DNA packaging machines. Proc. Natl. Acad. Sci. USA.

[48] Quiles-Puchalt N., Carpena N., Alonso J.C., Novick R.P., Marina A., Penadés J.R. (2014). Staphylococcal pathogenicity island DNA packaging system involving *cos* -site packaging and phage-encoded HNH endonucleases. Proc. Natl. Acad. Sci. USA.

[49] Duda R.L., Martincic K., Hendrix R.W. (1995). Genetic basis of bacteriophage HK97 prohead assembly. J. Mol. Biol..

[50] Bose B., Auchtung J.M., Lee C.A., Grossman A.D. (2008). A conserved anti-repressor controls horizontal gene transfer by proteolysis. Mol. Microbiol..

[51] Ludanyi M., Blanchard L., Dulermo R., Brandelet G., Bellanger L., Pignol D., Lemaire D., de Groot A. (2014). Radiation response in *Deinococcus deserti*: IrrE is a metalloprotease that cleaves repressor protein DdrO. Mol. Microbiol..

[52] Vujicic-Zagar A., Dulermo R., Le Gorrec M., Vannier F., Servant P., Sommer S., de Groot A., Serre L. (2009). Crystal structure of the IrrE protein, a central regulator of DNA damage repair in deinococcaceae. J. Mol. Biol..

[53] Vera M., Pagliai F., Guiliani N., Jerez C.A. (2008). The Chemolithoautotroph *Acidithiobacillus ferrooxidans* Can Survive under Phosphate-Limiting Conditions by Expressing a C-P Lyase Operon that Allows It to Grow on Phosphonates. Appl. Environ. Microbiol..

[54] Clokie M.R.J., Mann N.H. (2006). Marine cyanophages and light. Environ. Microbiol..

[55] Li Y., Wang Z., Chen J., Ernst R.K., Wang X. (2013). Influence of Lipid A Acylation Pattern on Membrane Permeability and Innate Immune Stimulation. Mar. Drugs.

[56] Vreeland R.H., Rosenzweig W.D., Powers D.W. (2000). Isolation of a 250 million-year-old halotolerant bacterium from a primary salt crystal. Nature.

[57] Fu Y., Deng S., Liang L., Wu Y., Gao M. (2019). Complete genome sequence of the novel phage vB_BthS-HD29phi infecting *Bacillus thuringiensis*. Arch. Virol..

[58] Gillis A., Mahillon J. (2014). Phages Preying on *Bacillus anthracis*, *Bacillus cereus*, and *Bacillus thuringiensis*: Past, Present and Future. Viruses.

[59] Seed K.D. (2015). Battling Phages: How Bacteria Defend against Viral Attack. PLOS Pathog..

[60] Tock M.R., Dryden D.T. (2005). The biology of restriction and anti-restriction. Curr. Opin. Microbiol..

[61] Bragg J.G., Chisholm S.W. (2008). Modeling the Fitness Consequences of a Cyanophage-Encoded Photosynthesis Gene. PLoS ONE.

[62] Mann N.H., Cook A., Millard A., Bailey S., Clokie M. (2003). Bacterial photosynthesis genes in a virus. Nature.

[63] Breitbart M., Thompson L., Suttle C., Sullivan M. (2007). Exploring the Vast Diversity of Marine Viruses. Oceanography.

[64] Thompson L.R., Zeng Q., Kelly L., Huang K.H., Singer A.U., Stubbe J., Chisholm S.W. (2011). Phage auxiliary metabolic genes and the redirection of cyanobacterial host carbon metabolism. Proc. Natl. Acad. Sci. USA.

[65] Lindell D., Jaffe J.D., Johnson Z.I., Church G.M., Chisholm S.W. (2005). Photosynthesis genes in marine viruses yield proteins during host infection. Nature.

[66] Pérez-Burgos M., García-Romero I., Jung J., Valvano M.A., Søgaard-Andersen L. (2019). Identification of the lipopolysaccharide O-antigen biosynthesis priming enzyme and the O-antigen ligase in Myxococcus xanthus: Critical role of LPS O-antigen in motility and development. Mol. Microbiol..

[67] Gross M., Marianovsky I., Glaser G. (2006). MazG–A regulator of programmed cell death in *Escherichia coli*. Mol. Microbiol..

[68] Duhaime M.B., Solonenko N., Roux S., Verberkmoes N.C., Wichels A., Sullivan M.B. (2017). Comparative Omics and Trait Analyses of Marine Pseudoalteromonas Phages Advance the Phage OTU Concept. Front. Microbiol..

[69] Mikoulinskaia G.V., Odinokova I.V., Zimin A.A., Lysanskaya V.Y., Feofanov S.A., Stepnaya O.A. (2009). Identification and characterization of the metal ion-dependent l-alanoyl-d-glutamate peptidase encoded by bacteriophage T5. FEBS J..

[70] Antonova N.P., Vasina D.V., Lendel A.M., Usachev E.V., Makarov V.V., Gintsburg A.L., Tkachuk A.P., Gushchin V.A. (2019). Broad bactericidal activity of the Myoviridae bacteriophage lysins LysAm24, LysECD7, and LysSi3 against Gram-negative ESKAPE pathogens. Viruses.

