# Supplementary material for: Genomic Characterization of a Halovirus Representing a Novel Siphoviral Cluster
Source: Viruses. 2023 Jun 19;15(6):1392. doi: 10.3390/v15061392 (PMC10305713; doi:10.3390/v15061392)
Supplement: Supplementary file 1 [file viruses-15-01392-s001.zip › viruses-2410709-supplementary.pdf]

Table S1 Genes annotation of phage YPHTV-1 (E<10^{-5})

ORF	Strand	Start	End	Length (bp)	Identity (%)	bitscore	*E*-value	Predicted function
1	-	101	1237	1137	99.47	159	4e-42	site-specific integrase
2	-	1246	1707	462	99.35	142	1e-40	ImmA/IrrE family metalloendopeptidase
3	-	1719	2030	312	96.12	206	1e-66	helix-turn-helix domain-containing protein
4	+	2182	2436	255	97.62	174	8e-55	helix-turn-helix domain-containing protein
5	+	2417	2689	273	97.78	184	e-58	group-specific protein
6	+	2686	2901	216	97.18	142	2e-42	hypothetical protein
7	+	2902	3147	246	67.9	126	6e-36	hypothetical protein
8	+	3245	3364	120	71.79	65.5	9e-13	hypothetical protein
9	+	3452	4960	1509	95.62	974	0	AAA family ATPase
10	+	4960	5229	270	61.63	108	2e-28	AbrB/MazE/SpoVT family DNA-binding domain-containing protein
11	+	5229	5840	612	100	407	4e-143	hypothetical protein
12	+	5840	6748	909	73.77	461	6e-161	recombinase RecT
13	+	6870	7166	297	94.19	170	7e-53	hypothetical protein
14	+	7135	7875	741	78.26	384	1e-132	MBL fold metallohydrolase
15	+	7876	8208	333	54.55	128	1e-53	hypothetical protein
16	+	8228	8998	771	96.09	509	0	replication protein
17	+	8976	10262	1287	66.9	613	0	AAA family ATPase
18	+	10455	10763	309	70.3	150	1e-44	MazG-like family protein
19	+	10760	11284	525	97.13	347	2e-120	hypothetical protein
20	+	11412	11876	465	99.35	304	6e-104	hypothetical protein
21	+	11928	12377	450	100	306	1e-104	NrdR transcription factor
22	+	12736	12927	192	100	126	2e-36	hypothetical protein
23	+	12927	13031	105	-	-	-	No hits
24	+	13034	13363	330	73.64	158	1e-47	hypothetical protein
25	+	13363	13743	381	94.44	221	5e-72	HNH endonuclease
26	+	13847	14290	444	87.76	273	7e-92	terminase small subunit
27	+	14296	16023	1728	98.78	1179	0	terminase large subunit
28	+	16004	16360	357	97.2	219	1e-71	hypothetical protein
29	+	16464	17621	1158	99.74	774	0	portal protein
30	+	17593	18174	582	99.48	391	5e-137	HK97 family phage prohead protease
31	+	18181	19545	1365	97.78	892	0	phage major capsid protein
32	+	19560	19835	276	95.6	184	2e-58	phage gp6-like head-tail connector protein
33	+	19852	20139	288	88.42	167	1e-51	hypothetical protein
34	+	20129	20461	333	95.45	220	5e-72	hypothetical protein
35	+	20454	20834	381	96.83	249	5e-83	HK97 gp10 family phage protein
36	+	20840	21457	618	88.29	380	4e-132	phage tail protein
37	+	21534	21716	183	96.67	101	4e-26	hypothetical protein
38	+	21730	22119	390	93.8	249	8e-83	hypothetical protein
39	+	22203	22343	141	97.83	95.9	2e-27	hypothetical protein
40	+	22359	25277	2919	89.01	1695	0	phage tail tape measure protein
41	+	25274	26080	807	60	331	5e-111	phage tail family protein
42	+	26093	28279	2187	96.7	1457	0	hypothetical protein
43	+	28422	29609	1188	73.16	602	0	O-antigen ligase family protein
44	+	29638	30678	1041	97.11	673	0	acyltransferase
45	+	30729	32720	1992	66.33	926	0	phage tail protein
46	+	32720	32926	207	95.59	135	1e-39	hypothetical protein

47	+	32919	33110	192	98.41	123	3e-35	hypothetical protein
48	+	33206	33382	177	84.48	101	2e-26	hypothetical protein
49	+	33398	33568	171	-	-	–	No hits
50	+	33719	34057	339	83.04	191	2e-60	hypothetical protein
51	+	34037	34342	270	65.12	120	4e-33	hypothetical protein
52	+	34344	35183	840	96.42	561	0	M15 family metallopeptidase
53	-	35213	35443	231	88.16	173	2e-40	hypothetical protein
54	-	35503	35850	348	90.27	219	1e-71	hypothetical protein
55	-	36054	36272	219	100	130	7e-38	helix-turn-helix domain-containing protein
56	+	36478	37275	789	98.87	547	0	DUF87 domain-containing protein
57	+	37277	37747	471	97.44	371	7e-109	hypothetical protein

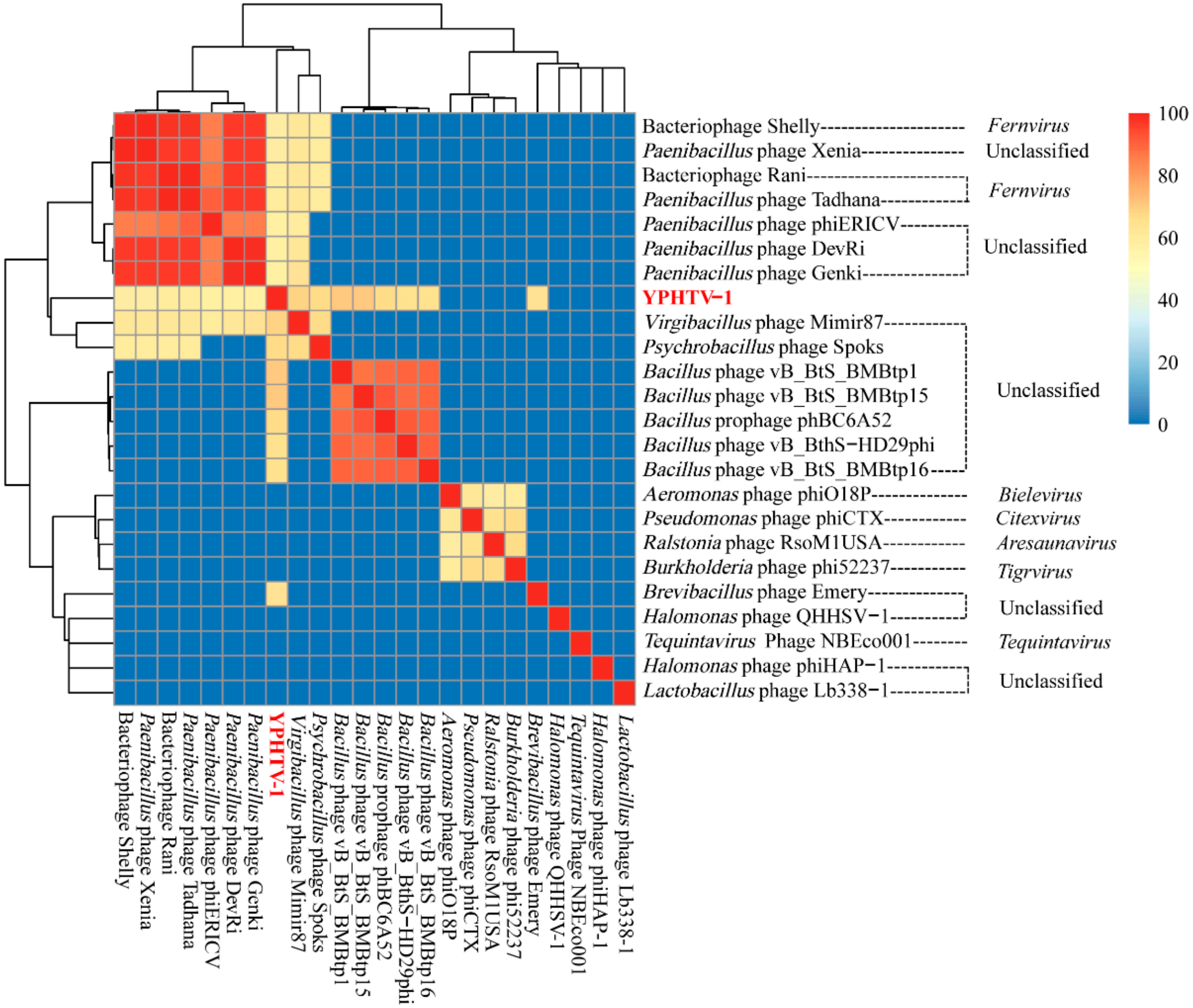

Figure S1. Phage genome ANI heatmap. The horizontal and vertical coordinates are the phage names, and the color of the intersecting squares represents the ANI value of the two phages.

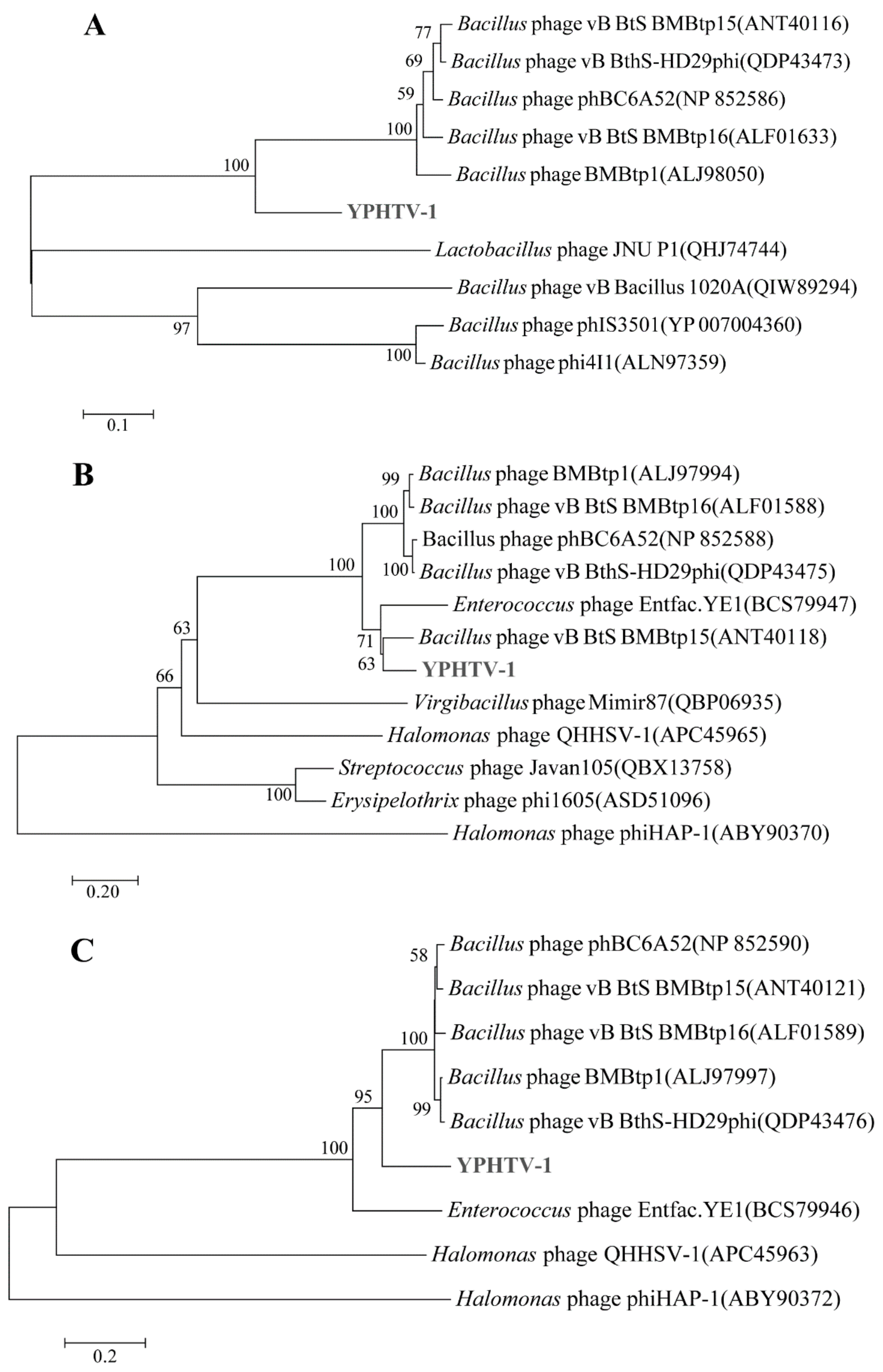
A
77
69
59
100
100
97
100
Bacillus phage vB BtS BMBtp15(ANT40116)
Bacillus phage vB BthS-HD29phi(QDP43473)
Bacillus phage phBC6A52(NP 852586)
Bacillus phage vB BtS BMBtp16(ALF01633)
Bacillus phage BMBtp1(ALJ98050)
YPHTV-1
Lactobacillus phage JNU P1(QHJ74744)
Bacillus phage vB Bacillus 1020A(QIW89294)
Bacillus phage phIS3501(YP 007004360)
Bacillus phage phi4I1(ALN97359)
0.1
B
99
100
100
100
63
66
71
63
100
Bacillus phage BMBtp1(ALJ97994)
Bacillus phage vB BtS BMBtp16(ALF01588)
Bacillus phage phBC6A52(NP 852588)
Bacillus phage vB BthS-HD29phi(QDP43475)
Enterococcus phage Entfac.YE1(BCS79947)
Bacillus phage vB BtS BMBtp15(ANT40118)
YPHTV-1
Virgibacillus phage Mimir87(QBP06935)
Halomonas phage QHHSV-1(APC45965)
Streptococcus phage Javan105(QBX13758)
Erysipelothrix phage phi1605(ASD51096)
Halomonas phage phiHAP-1(ABY90370)
0.20
C
58
100
99
95
100
Bacillus phage phBC6A52(NP 852590)
Bacillus phage vB BtS BMBtp15(ANT40121)
Bacillus phage vB BtS BMBtp16(ALF01589)
Bacillus phage BMBtp1(ALJ97997)
Bacillus phage vB BthS-HD29phi(QDP43476)
YPHTV-1
Enterococcus phage Entfac.YE1(BCS79946)
Halomonas phage QHHSV-1(APC45963)
Halomonas phage phiHAP-1(ABY90372)
0.2

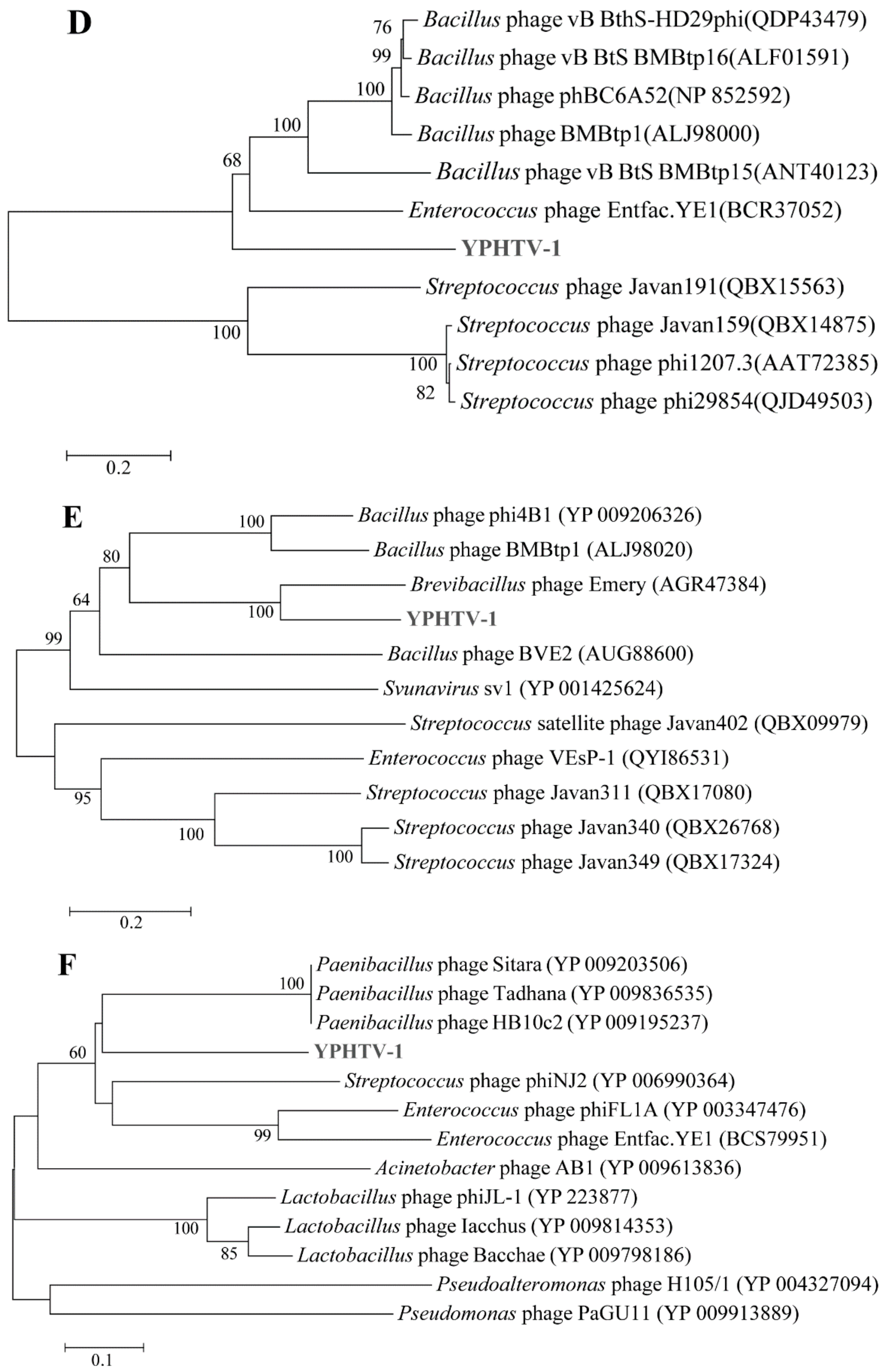

Figure S2. The amino acid sequence phylogenetic trees based on HNH endonuclease (A), terminase large subunit (B), portal protein (C), major capsid protein (D), site-specific integrase (E), and MazG-like family protein (F) of YPHTV-1 and other phages. Phylogenetic trees were constructed using the neighbor-joining method by Mega 7.0. All parameters are default except the Bootstrap value is 1000, the p-distance model is used to calculate the
